# Restoration of pre-arthritic coronal alignment improves joint perception following fixed-bearing medial unicompartmental knee arthroplasty

**DOI:** 10.3389/fsurg.2026.1807125

**Published:** 2026-05-20

**Authors:** Yonggang Peng, Jia Chen, Feng Zhao, Gang Ji, Chaohua Zhu, Guobin Liu

**Affiliations:** Department of Orthopedics, The First Hospital of Hebei Medical University, Shijiazhuang, China

**Keywords:** coronal alignment, forgotten joint score, patient-reported outcomes, pre-arthritic alignment, unicompartmental knee arthroplasty

## Abstract

**Background:**

The optimal postoperative alignment strategy in unicompartmental knee arthroplasty (UKA) remains a topic of debate. Although traditional mechanical alignment targets a neutral axis, restoring a patient's pre-arthritic, constitutional alignment may enhance clinical outcomes and joint perception. This study compares patient-reported outcomes (PROs) and joint awareness between patients who achieved pre-arthritic alignment and those who did not after medial fixed-bearing UKA.

**Methods:**

In this retrospective cohort study, 266 patients who underwent medial fixed-bearing UKA between January, 2022 and May, 2023 were included, with a mean follow-up of 3.5 years. Pre-arthritic coronal alignment was estimated using the arithmetic Hip-Knee-Ankle angle (aHKA). Preoperative and postoperative coronal alignment measurements were performed using weight-bearing long-leg radiographs. Patients were stratified into two groups: pre-arthritically aligned (postoperative mechanical Hip-Knee-Ankle angle [mHKA] within ±3° of aHKA) and nonpre-arthritically aligned (mHKA beyond ±3° of aHKA). PROs assessed at final follow-up included Oxford Knee Score (OKS), Forgotten Joint Score-12 (FJS-12), and Kujala Score.

**Results:**

Among the cohort, 212 knees (79.7%) were classified as pre-arthritically aligned and 54 (20.3%) as nonpre-arthritically Aligned. The groups were comparable in all baseline demographic and radiological parameters (*p* > 0.05). However, the pre-arthritically aligned group demonstrated superior PROs: OKS (41.3 ± 5.4 vs. 39.2 ± 5.9, *p* < 0.05), FJS-12 (78.9 ± 2.3 vs. 71.1 ± 3.2, *p* < 0.05), and Kujala Score (85.4 ± 6.0 vs. 81.3 ± 5.5, *p* < 0.05).

**Conclusion:**

Restoring pre-arthritic coronal alignment following fixed-bearing medial UKA is associated with superior patient-reported outcomes, including significantly better joint perception and patellofemoral function compared to nonpre-arthritic alignment. Further long-term follow-up is required to confirm the durability of these findings.

## Introduction

Unicompartmental knee arthroplasty (UKA) is an established and effective treatment for isolated anteromedial osteoarthritis (OA) of the knee (1). Compared to total knee arthroplasty, UKA offers distinct advantages, including reduced intraoperative blood loss, faster postoperative recovery, and preservation of native knee kinematics by retaining both cruciate ligaments and the intact lateral compartment cartilage (2).

Appropriate postoperative lower limb alignment is a critical factor influencing long-term implant survivorship and functional outcomes following UKA (3, 4). However, there is still no consensus regarding the optimal alignment target. The conventional mechanical alignment strategy aims to achieve a neutral mechanical axis postoperatively (5), but this standardized approach may overlook significant inter-individual anatomical variations (6). Consequently, individualized alignment strategies that aim to recreate a patient's unique pre-arthritic alignment, the constitutional alignment present before the onset of OA, have gained traction (7). The arithmetic Hip-Knee-Ankle angle (aHKA) has been validated as a reliable method to estimate this constitutional alignment in patients with arthritis (8, 9), and emerging evidence suggests that patients whose postoperative alignment falls within a narrow margin (±3°) of their pre-arthritic alignment achieve superior joint function (10, 11).

However, date specifically linking restoration of pre-arthritic alignment to joint perception (Forgotten Joint Score-12) in fixed-bearing UKA is limited. Therefore, this study was conducted to investigate the impact of restoring pre-arthritic coronal alignment on patient-reported outcomes and achievement of this forgotten joint state in patients undergoing fixed-bearing medial UKA specifically.

## Materials and methods

Following approval from the Institutional Review Board, a retrospective cohort study was performed on consecutive patients who underwent fixed-bearing medial UKA at our institution between January, 2022 and May, 2023. The indications for UKA included: (1) isolated anteromedial knee OA, (2) intact anterior and posterior cruciate ligaments, (3) flexion contracture <10°, and (4) varus deformity <15° correctible under valgus stress. The exclusion criteria were: (1) patients did not have adequate preoperative or postoperative radiographic series, (2) follow-up duration of less than two years, (3) history of femoral or tibial fracture on either side. (4) previous knee surgery (excluding diagnostic arthroscopy); (5) inflammatory arthritis or post-traumatic arthritis. Based on these criteria, 266 patients were enrolled for whom the median follow-up period was 3.5 years.

### Radiographic evaluation

**Figure 1:**
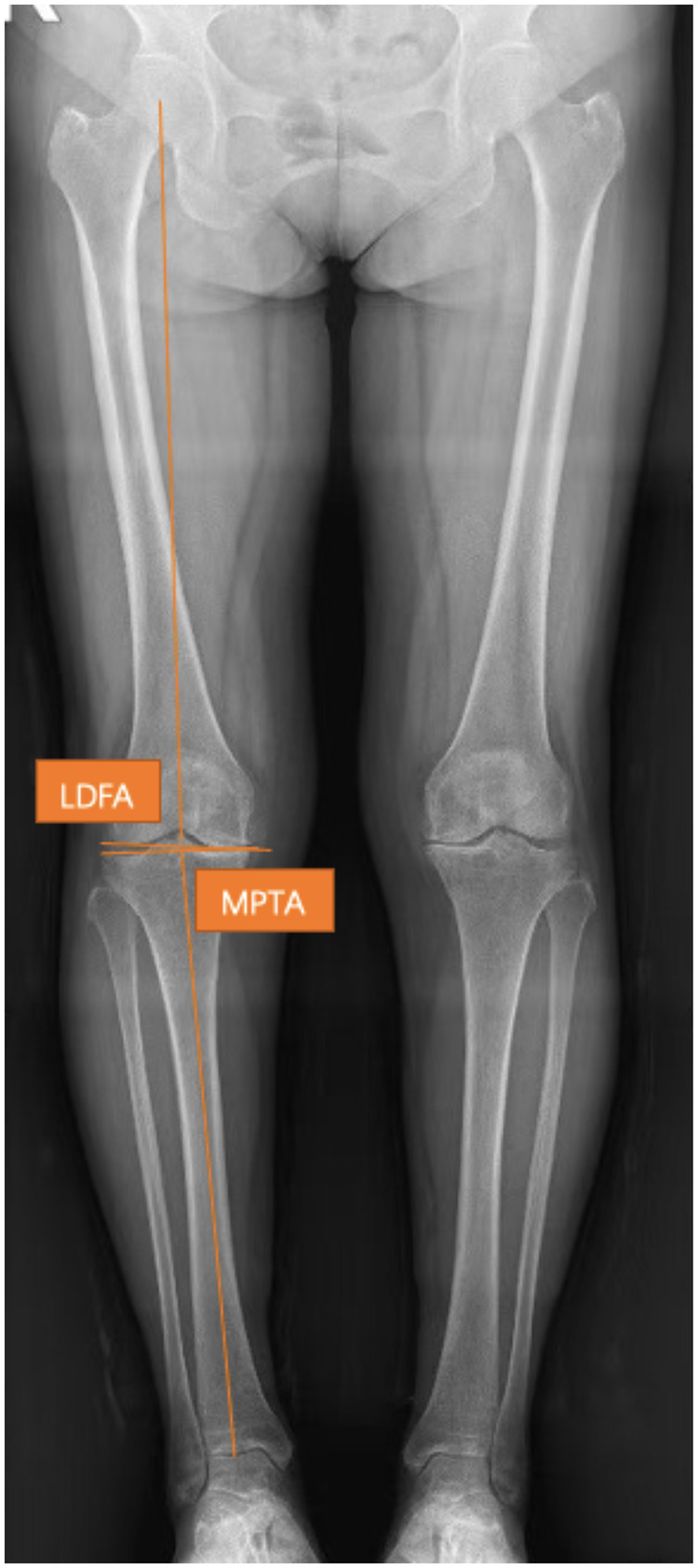
Preoperative long-leg radiograph. The lateral distal femoral angle (LDFA) was defined as the lateral angle formed by the femoral mechanical axis and the joint line of the distal femur. The medial proximal tibial angle (MPTA) was defined as the medial angle formed by the tibial mechanical axis and the joint line of the proximal tibia. The arithmetic hip-knee-ankle angle (aHKA) was calculated using the formula 180° − LDFA + MPTA.

All long-leg radiographs were obtained with the patient standing in a standardized weight-bearing position: feet placed at shoulder-width apart, patellae facing forward, and weight equally distributed on both lower limbs. Preoperative radiographs were obtained prior to surgery, and postoperative radiographs were obtained at the second day after surgery. Preoperative standard weight-bearing long-leg radiographs were used to measure the mechanical Hip-Knee-Ankle angle (mHKA), the medial proximal tibial angle (MPTA), and the lateral distal femoral angle (LDFA) (8). The constitutional, pre-arthritic alignment was retrospectively estimated for the operative knee using the arithmetic HKA (aHKA) algorithm: aHKA = 180° − LDFA + MPTA (Figure 1) (9). Postoperative mHKA was measured on the postoperative weight-bearing long-leg radiograph. A knee was considered “pre-arthritically aligned” if the postoperative mHKA was restored within ±3.0° of the calculated aHKA (11). Accordingly, patients were divided into two groups: pre-arthritically aligned (postoperative mHKA restored within ±3.0° of the aHKA) and nonpre-arthritically aligned (postoperative mHKA restored beyond ±3.0° of the aHKA) (11). All angular measurements (mHKA, LDFA, MPTA) were performed using a digital PACS system, with measurements taken by two independent observers as described previously.

### Outcome evaluation

Patient-reported outcomes including Oxford Knee Score (OKS) (12), Forgotten Joint Score (13), and Kujula Score (14) were collected postoperatively at the time of the latest follow-up.

### Statistical analysis

All statistical analysis was performed using SPSS software (Version 26.0, IBM Corp). The normality of continuous variables was assessed using the Shapiro–Wilk test. Continuous data were expressed as mean ± standard deviation (SD). An independent samples *t*-test was used to compare PROs between the two alignment groups. Non-normally distributed continuous variables were analyzed using the Mann–Whitney U test. Categorical variables are presented as frequency and percentage, with intergroup comparisons conducted using the chi-square test or Fisher's exact test, as appropriate, where a *p*-value of <0.05 was considered to indicate a statistically significant result.

## Results

### Patient demographics and radiological parameters

A total of 266 patients were included in the study, with a mean age of 64.7 ± 7.3 years and a mean BMI of 26.5 ± 3.2 kg/m^2^. The majority of patients were female (*n* = 199, 74.8%), and the mean preoperative mHKA was 172.2° ± 3.1°. Postoperatively, the mean mHKA was 177.3° ± 2.9°. Other radiological parameters are summarized in Table 1.

**Table 1:** Patient demographic and radiological parameters.

Variable	Total (266)
Age	64.7 ± 7.3
Sex (*n*)	
Female	199
Male	67
BMI	26.5 ± 3.2
Preoperative mHKA(°)	172.2 ± 3.1
MPTA(°)	84.7 ± 2.3
LDFA(°)	88.5 ± 1.9
aHKA(°)	177.1 ± 2.1
Postoperative mHKA(°)	177.3 ± 2.9

BMI, body mass index; aHKA, arithmetic hip-knee-ankle angle; LDFA, lateral distal femoral angle; MPTA, medial proximal tibial angle; mHKA, mechanical hip-knee-ankle angle.

### Comparison based on pre-arthritic alignment

Based on the defined criterion, the pre-arthritically aligned group comprised 212 patients (79.7%), and the non-pre-arthritically aligned group included 54 patients (20.3%). The two groups were comparable in all baseline demographic and radiological parameters. However, statistically significant differences were observed in all PROMs at final follow-up (Table 2). Importantly, the pre-arthritically aligned group demonstrated superior outcomes in OKS, FJS-12, and Kujala Score.

**Table 2:** Patient demographic, radiographic, and PROM outcomes by alignment group.

Patient	Pre-arthritically aligned	Nonpre-arthritically aligned	*p* value
N	212 (79.7%)	54 (20.3%)	
Age(y)	64.3 ± 6.5	64.5 ± 6.8	n.s
Sex (*n*(%))			
Female	162 (76.4%)	37 (68.5%)	n.s
Male	50 (23.6%)	17 (31.5%)	n.s
Side (*n*(%))			
Left	112 (52.9%)	28 (51.9%)	n.s
Right	100 (47.1%)	26 (48.1%)	n.s
BMI	26.9 ± 2.6	27.1 ± 2.4	n.s
Preoperative mHKA (°)	173.2 ± 2.9	173.3 ± 3.1	n.s
LDFA (°)	88.4 ± 2.8	88.3 ± 3.0	n.s
MPTA (°)	84.9 ± 2.5	85.7 ± 2.3	n.s
aHKA (°)	177.5 ± 2.1	178.1 ± 4.9	n.s
Postoperative mHKA (°)	177.6 ± 2.4	177.9 ± 2.9	n.s
OKS	41.3 ± 5.4	39.2 ± 5.9	<0.05
FJS-12	78.9 ± 2.3	71.1 ± 3.2	<0.05
Kujala	85.4 ± 6.0	81.3 ± 5.5	<0.05

Outcomes are presented by alignment group as mean ± standard deviation (SD) or in numbers with the frequency in parentheses.

aHKA, arithmetic hip-knee-ankle angle; LDFA, lateral distal femoral angle; MPTA, medial proximal tibial angle; mHKA, mechanical hip-knee-ankle angle; n.s, not significant.

## Discussion

The principal finding of this study is that successful restoration of pre-arthritic coronal alignment is associated with significantly better patient-reported outcomes following fixed-bearing medial UKA. Specifically, patients in the pre-arthritically aligned group achieved superior Forgotten Joint score-12 outcomes, indicating a higher likelihood of experiencing a “forgotten joint” state. Additionally, they demonstrated better patellofemoral function as measured by the Kujala Score.

The debate over ideal postoperative alignment in UKA has evolved from a binary choice between neutral mechanical alignment and residual varus toward a more nuanced, individualized paradigm. Traditional mechanical alignment, which aims for a neutral mechanical axis (mHKA = 180° ± 3°), has been the historical standard; however, this approach may inadvertently overcorrect patients with constitutional varus (15). Several studies have investigated the optimal degree of residual varus. Zuiderbaan et al., in a cohort of 104 patients receiving medial fixed-bearing UKA, reported that the highest postoperative WOMAC scores correlated with a residual varus alignment of 1°–4° (16). Similarly, a study by Wang et al. involving 302 cases of medial fixed-bearing UKA identified a postoperative varus of 1.5°–4° as correlating with the highest likelihood of achieving a “forgotten joint” state (17). Conversely, Vasso et al. found superior functional results in patients with a varus alignment of 5°–7° after medial fixed-bearing UKAs (18). These discrepancies suggest that a single numeric target may not be universally applicable, highlighting the need for an individualized approach.

The concept of pre-arthritic alignment is grounded in the observation that constitutional varus is present in a substantial proportion of the healthy population (15). For these individuals, achieving a neutral axis might overcorrect their native alignment, which could consequently compromise knee kinematics and result in an unnatural sensation. For example, Hernigou et al. found that overcorrecting the varus deformity was associated with a higher risk of degeneration in the lateral compartment and that residual varus deformity may lead to an increased risk of tibial polyethylene wear (19).

Therefore, some researchers suggest personalized UKA alignment by measuring the aHKA to restore the patient's pre-arthritic alignment (8). The arithmetic HKA (aHKA) method, first described by MacDessi et al., provides a reliable estimate of constitutional coronal alignment in arthritic knees by combining the lateral distal femoral angle (LDFA) and the medial proximal tibial angle (MPTA) (8). This approach avoids reliance on the contralateral limb and has been validated against normal populations (9). Our high success rate (79.7%) in achieving the planned pre-arthritic alignment target is consistent with previous reports, such as the study by Plancher et al. where 85% of patients achieved similar alignment (11). This reproducibility underscores the feasibility of implementing a pre-arthritic alignment strategy in clinical practice.

The concept of pre-arthritic alignment is closely related to the recently proposed Coronal Plane Alignment of the Knee (CPAK) classification system. CPAK categorizes knees based on two parameters: the arithmetic HKA (aHKA), which estimates constitutional alignment, and the joint line obliquity (JLO), calculated as the difference between LDFA and MPTA (20). While CPAK provides a framework for describing knee phenotypes, our study specifically focused on the restoration of aHKA—the constitutional coronal alignment—rather than the maintenance of the full CPAK phenotype.

A recent study by Sahbat et al. (21) reported that maintaining CPAK classification after medial UKA did not significantly impact patient-reported outcomes or implant survival. Although these findings may appear to contrast with ours, several distinctions are noteworthy. First, Sahbat et al. assessed whether CPAK type remained unchanged postoperatively, whereas we evaluated restoration of aHKA within ±3° of constitutional alignment—a more individualized target. Second, we employed FJS-12 and Kujala Score, which are more sensitive to subtle alignment-related improvements than general knee function scores. Third, our cohort explicitly targeted pre-arthritic alignment. Thus, while maintaining CPAK classification may not be necessary for acceptable general outcomes, restoring constitutional coronal alignment may further optimize joint perception and patellofemoral function. These studies are complementary rather than contradictory. Similarly, Kim et al. (22) reported that preserving CPAK classification after medial UKA correlated with superior patient-reported outcomes. However, their follow-up duration was shorter (mean 2 years) than that of Sahbat et al. and our study (mean 3.5 years). The variability in findings across studies underscores the need for longer-term follow-up and consistent outcome measures.

Furthermore, our use of the FJS-12, a sensitive tool for detecting differences in joint awareness among well-performing patients, provides strong evidence that pre-arthritic alignment enhances the “forgotten joint” experience (23). The forgotten joint is regarded as the ultimate goal of joint arthroplasty, representing a highly functional state that may be related to proper postoperative ligament tension (24). Improved ligament balance and joint congruency are plausible mechanisms for this enhanced perception (25). Wang et al. similarly reported that achieving postoperative alignment within ±2° of aHKA led to higher Forgotten Joint Scores in a cohort of 244 medial UKAs (6), corroborating our findings and supporting the clinical relevance of this alignment target.

Our results show that restoration of pre-arthritic coronal alignment was also associated with significantly higher Kujala scores, which is also consistent with other research findings. For instance, in a study of 618 knees with robotic-assisted medial UKA (minimum 2-year follow-up), Vossen et al. demonstrated a significant correlation: preserved CPAK phenotypes and restored pre-arthritic coronal alignment were linked to superior Kujala scores compared to altered phenotypes (26). Moreover, a study of 537 medial fixed-bearing UKAs by Bayoumi et al. revealed a correlation between under-correction from the pre-arthritic alignment and lower postoperative Kujala scores (7). Another study found that knees with under correction from their pre-arthritic coronal alignment after medial UKA were related to a two-fold higher probability of having postoperative patellofemoral incongruence (27). The greater frequency of patellofemoral incongruence among under corrected knees could be a consequence of under correction of the mechanical axis, potentially bringing about increased contact forces across the patellofemoral joint (27). Micicoi et al. further emphasized that restoring preoperative tibial alignment improves functional results after medial UKA, reinforcing the importance of respecting native anatomy (28).

The biomechanical rationale for restoring pre-arthritic alignment is compelling. Restoring the native joint line obliquity and soft-tissue envelope tension likely optimizes load distribution across the medial compartment and the patellofemoral joint (7, 11, 29). Rivière et al., in a systematic review, concluded that kinematic alignment in UKA is safe and may improve functional outcomes by preserving native knee kinematics (30). Our findings align with this philosophy, extending the evidence base to specifically support restoration of constitutional coronal alignment as a key surgical target.

Despite these results, this study has several limitations. First, its retrospective design carries inherent risks of selection and information bias. Second, it included only fixed-bearing medial UKA, so the results may not apply to mobile-bearing medial UKA or lateral UKA. Third, it lacks long-term follow-up data on implant survivorship, which is necessary to confirm its impact on prosthesis survival.

## Conclusion

We find that restoring pre-arthritic coronal alignment during fixed-bearing medial UKA is a reproducible strategy that is also associated with superior patient-reported outcomes, including significantly better joint perception and patellofemoral function, compared to nonpre-arthritic alignment. Further long-term follow-up is required to confirm the durability of these findings.

## Data Availability

The raw data supporting the conclusions of this article will be made available by the authors, without undue reservation.

## References

[1] Liddle AD Pandit H Judge A Murray DW. Patient-reported outcomes after total and unicompartmental knee arthroplasty: a study of 14,076 matched patients from the national joint registry for England and Wales. Bone Joint J. (2015) 97-b(6):793–801. 10.1302/0301-620X.97B6.35155 26033059

[2] Bayoumi T Van Der List JP Ruderman LV Zuiderbaan HA Kerkhoffs GMMJ Pearle AD. Successful same-day discharge in 88% of patients after unicompartmental knee arthroplasty: a systematic review and meta-analysis. Knee Surg Sports Traumatol Arthrosc. (2023) 31(3):946–62. 10.1007/s00167-022-07094-0 35951077 PMC9366132

[3] Ishibashi K Sasaki E Otsuka H Kazushige K Yamamoto Y Ishibashi Y. Valgus correctability and meniscal extrusion were associated with alignment after unicompartmental knee arthroplasty. Clin Orthop Relat Res. (2020) 478(7):1636–44. 10.1097/CORR.0000000000001260 32324667 PMC7310375

[4] Slaven SE Cody JP Sershon RA Ho H Hopper RH Fricka KB The impact of coronal alignment on revision in medial fixed-bearing unicompartmental knee arthroplasty. J Arthroplasty. (2020) 35(2):353–7. 10.1016/j.arth.2019.09.038 31668526

[5] Whiteside LA. Making your next unicompartmental knee arthroplasty last: three keys to success. J Arthroplasty. (2005) 20(4 Suppl 2):2–3. 10.1016/j.arth.2005.03.029 15991117

[6] Wang Z Deng W Zhou Y Huang Y Guo S Zhang Y. Restoring prearthritic alignment improves joint perception in medial unicompartmental knee arthroplasty. J Exp Orthop. (2025) 12(3):e70389. 10.1002/jeo2.70389 40766802 PMC12322694

[7] Bayoumi T Burger JA Ruderman LV van der List JP Zuiderbaan HA Kerkhoffs GMMJ Restoration or relative overcorrection of pre-arthritic coronal alignment leads to improved results following medial unicompartmental knee arthroplasty. Knee Surg Sports Traumatol Arthrosc. (2023) 31(9):3981–91. 10.1007/s00167-023-07441-9 37145133

[8] MacDessi SJ Griffiths-Jones W Harris IA Bellemans J Chen DB. The arithmetic HKA (aHKA) predicts the constitutional alignment of the arthritic knee compared to the normal contralateral knee: a matched-pairs radiographic study. Bone Jt Open. (2020) 1(7):339–45. 10.1302/2633-1462.17.BJO-2020-0037.R1 33215122 PMC7659698

[9] Griffiths-Jones W Chen DB Harris IA Bellemans J MacDessi SJ. Arithmetic hip-knee-ankle angle (aHKA): an algorithm for estimating constitutional lower limb alignment in the arthritic patient population. Bone Jt Open. (2021) 2(5):351–8. 10.1302/2633-1462.25.BJO-2021-0028.R1 34042492 PMC8168548

[10] Zhao Y Liang X Wang Y Lu X Lu C Xu Y. Fixed-bearing medial unicompartmental knee arthroplasty restores pre-arthritic coronal alignment and achieves satisfactory functional outcomes: a retrospective study. J Orthop Surg Res. (2024) 19(1):676. 10.1186/s13018-024-05168-y 39428486 PMC11492782

[11] Plancher KD Brite JE Briggs KK Petterson SC. Pre-arthritic/kinematic alignment in fixed-bearing medial unicompartmental knee arthroplasty results in return to activity at mean 10-year follow-up. J Bone Joint Surg Am. (2022) 104(12):1081–9. 10.2106/JBJS.21.00801 36149243

[12] Dawson J Fitzpatrick R Murray D Carr A. Questionnaire on the perceptions of patients about total knee replacement. J Bone Joint Surg Br. (1998) 80(1):63–9. 10.1302/0301-620X.80B1.0800063 9460955

[13] Thienpont E Opsomer G Koninckx A Houssiau F. Joint awareness in different types of knee arthroplasty evaluated with the forgotten joint score. J Arthroplasty. (2014) 29(1):48–51. 10.1016/j.arth.2013.04.024 23688851

[14] Kujala UM Jaakkola LH Koskinen SK Taimela S Hurme M Nelimarkka O. Scoring of patellofemoral disorders. Arthroscopy. (1993) 9(2):159–63. 10.1016/S0749-8063(05)80366-4 8461073

[15] Bellemans J Colyn W Vandenneucker H Victor J. The chitranjan ranawat award: is neutral mechanical alignment normal for all patients? The concept of constitutional varus. Clin Orthop Relat Res. (2012) 470(1):45–53. 10.1007/s11999-011-1936-5 21656315 PMC3237976

[16] Zuiderbaan HA Van Der List JP Chawla H Khamaisy S Thein R Pearle AD. Predictors of subjective outcome after medial unicompartmental knee arthroplasty. J Arthroplasty. (2016) 31(7):1453–8. 10.1016/j.arth.2015.12.038 26928182

[17] Wang Z Deng W Shao H Zhou Y Yang D Li H. Predictors of a forgotten joint after medial fixed-bearing unicompartmental knee arthroplasty. Knee. (2022) 37:103–11. 10.1016/j.knee.2022.06.004 35753203

[18] Vasso M Del Regno C D'amelio A Viggiano D Corona K Schiavone Panni A. Minor varus alignment provides better results than neutral alignment in medial UKA. Knee. (2015) 22(2):117–21. 10.1016/j.knee.2014.12.004 25659441

[19] Hernigou P Deschamps G. Alignment influences wear in the knee after medial unicompartmental arthroplasty. Clin Orthop Relat Res. (2004) 423:161–5. 10.1097/01.blo.0000128285.90459.12 15232443

[20] Macdessi SJ Griffiths-Jones W Harris IA Bellemans J Chen DB. Coronal plane alignment of the knee (CPAK) classification. Bone Joint J. (2021) 103-b(2):329–37. 10.1302/0301-620X.103B2.BJJ-2020-1050.R1 33517740 PMC7954147

[21] Sahbat Y Gulagaci F Mabrouk A Karam KM Jacquet C Ollivier M Maintenance of coronal alignment and joint line obliquity has no effect on unicompartmental knee arthroplasty: clinical results at five years follow-up. J Arthroplasty. (2025) 40(6):1508–15.e1. 10.1016/j.arth.2024.11.041 39586406

[22] Kim SE Yun KR Lee JM Han HS. Preserving coronal knee alignment of the knee (CPAK) in unicompartmental knee arthroplasty correlates with superior patient-reported outcomes. Knee Surg Relat Res. (2024) 36(1):1. 10.1186/s43019-023-00204-3 38167246 PMC10763258

[23] Behrend H Giesinger K Giesinger JM Kuster MS. The “forgotten joint” as the ultimate goal in joint arthroplasty: validation of a new patient-reported outcome measure. J Arthroplasty. (2012) 27(3):430–6.e1. 10.1016/j.arth.2011.06.035 22000572

[24] Kamenaga T Muratsu H Kanda Y Miya H Kuroda R Matsumoto T. The influence of postoperative knee stability on patient satisfaction in cruciate-retaining total knee arthroplasty. J Arthroplasty. (2018) 33(8):2475–9. 10.1016/j.arth.2018.03.017 29656976

[25] Shin KH Jang KM Han SB. Residual varus alignment can reduce joint awareness, restore joint parallelism, and preserve the soft tissue envelope during total knee arthroplasty for varus osteoarthritis. Knee Surg Sports Traumatol Arthrosc. (2022) 30(2):507–16. 10.1007/s00167-020-06201-3 32743784

[26] Vossen RJM Burger JA ten Noever de Brauw GV Bayoumi T Fiore JA Ruderman LV Preservation of prearthritic coronal knee phenotype and prearthritic coronal alignment yielded improved Kujala scores following ligament-guided medial unicompartmental knee arthroplasty. Knee Surg Sports Traumatol Arthrosc. (2024) 32(12):3185–97. 10.1002/ksa.12282 38804655

[27] Ruderman LV Bayoumi T Burger JA Zuiderbaan HA Pearle AD. Higher incidence of patellar incongruence after under correction of pre-arthritic coronal alignment following medial unicompartmental knee arthroplasty. Knee Surg Sports Traumatol Arthrosc. (2023) 31(12):5773–82. 10.1007/s00167-023-07645-z 37934285

[28] Micicoi L Machado A Ernat J Schippers P Bernard de Dompsure R Bronsard N Restoration of preoperative tibial alignment improves functional results after medial unicompartmental knee arthroplasty. Knee Surg Sports Traumatol Arthrosc. (2023) 31(11):5171–9. 10.1007/s00167-023-07588-5 37758904

[29] Van Der List JP Sheng DL Kleeblad LJ Chawla H Pearle AD. Outcomes of cementless unicompartmental and total knee arthroplasty: a systematic review. Knee. (2017) 24(3):497–507. 10.1016/j.knee.2016.10.010 27923627

[30] Rivière C Sivaloganathan S Villet L Cartier P Lustig S Vendittoli PA Kinematic alignment of medial UKA is safe: a systematic review. Knee Surg Sports Traumatol Arthrosc. (2022) 30(3):1082–94. 10.1007/s00167-021-06462-6 33743031

